# Influence of the Halloysite Nanotube (HNT) Addition on Selected Mechanical and Biological Properties of Thermoplastic Polyurethane

**DOI:** 10.3390/ma14133625

**Published:** 2021-06-29

**Authors:** Maciej Mrówka, Małgorzata Szymiczek, Tomasz Machoczek, Mirosława Pawlyta

**Affiliations:** 1 Department of Theoretical and Applied Mechanics, Faculty of Mechanical Engineering, Silesian University of Technology, Konarskiego 18A Street, 44-100 Gliwice, Poland; malgorzata.szymiczek@polsl.pl (M.S.); tomasz.machoczek@polsl.pl (T.M.); 2 Materials Research Laboratory, Faculty of Mechanical Engineering, Silesian University of Technology, Konarskiego 18A Street, 44-100 Gliwice, Poland; miroslawa.pawlyta@polsl.pl

**Keywords:** biomaterial, nanocomposite, thermoplastic polyurethane (TPU), halloysite nanotubes (HNT), mechanical properties, biological properties, tribology, processing, 3D printing

## Abstract

Halloysite nanotube (HNT) additions to the thermoplastic polyurethane (TPU) system were thoroughly evaluated in this study. The resultant composites have been designed for future personalized intervertebral disc implant applications, which requires additional technology to obtain the appropriate geometry unique to each patient. These requirements can be fulfilled using 3D printing. In this work, a technology was developed to produce filaments for fused deposition modeling (FDM). Nanocomposites were prepared using variable HNT content (1, 2, and 3 wt.%). The nanostructure of the resultant composites was confirmed using scanning transmission electron microscopy (STEM). Mechanical tests were used to measure the tensile modulus, stress, and elongation the composites and TPU matrix. Nanocomposites with 2% HNT content were able to withstand 26% increased stress and 50% increased elongation compared to pure TPU before fracturing in addition to a 13% reduction in the friction coefficient. A MTT cytotoxicity assay confirmed the cytotoxicity of all tested materials against human epidermal keratinocyte cells (HaCaT).

## 1. Introduction

Due to their desirable physicochemical and mechanical properties and non-toxic nature, thermoplastic polyurethanes (TPUs) have been used in many industries, including medicine [1,2,3]. TPUs are used in the production of a wide variety of medical components such as artificial cardiac valves, artificial blood vessels, esophagus elements, breast implants, blood bags, extracorporeal blood oxygenation apparatuses, catheters, dental floss, oxygen mask elements, bone adhesives, stomach balloons, esophageal prostheses elements, and tendon prostheses [4,5,6,7]. Some studies report that TPU can also serve as a material for intervertebral disc prostheses [8,9]. One challenge in modern materials engineering is the development of new materials for medical applications [10,11]. Nanocomposites are a promising solution, as they have improved mechanical properties and are less abrasive in relation to the matrix material [12,13,14]. The addition of nanofillers in the PU matrix can be used to influence the physic, mechanical and biological material properties. For instance, adding metal nanoparticles, such as Ag, Au, ZnO_2_, TiO_2_, and NiO, to the PU matrix can improve their antibacterial activity and enhance thermal stability, mechanical, dynamic mechanical properties, and biostability [15,16,17,18]. Additionally, regenerated tissue in PU-5 wt.% grapheme (PU-G5) conduits showed an obvious response in the compound action potential (CAP) examination with a similar CAP wave pattern to that of the sciatic nerve. Scaffolding used in cardiac tissue engineering require thrombo-resistant and anticoagulant characteristics if they are to be used for cardiovascular applications. In this study, a new PU nanocomposite with carotino oil was produced via electro-spinning [15,19]. The developed PU/NiO nanocomposite delayed blood clotting time and lowered the hemolytic percentage, indicating improved anticoagulant characteristics compared with pristine PU. Patches made with the PU/NiO nanocomposite also rendered improved physicochemical compatibility, blood compatibility, and non-toxicity to fibroblast cells [15,20]. Similarly, a synthesized PU/TiO₂ patch exhibited improved physicochemical characteristics with enhanced blood compatibility parameters and cell viability rates, making it another promising candidate for cardiac tissue engineering [15,21]. Other PU nanocomposites have been successfully prepared using a bio-based hyper-branched PU and iron (III) oxide nanoparticles, which displayed magnetic behavior with enhanced biodegradation, biocompatibility, antimicrobial properties, and shape recovery effects compared with pristine PU [22,23]. Among the many available nanofillers are halloysite nanotubes (HNT), which are nanoparticles with a tubular structure that have already been successfully applied in controlled drug delivery systems. Conducted research has proven that this material is non-toxic to humans which, combined with its tubular structure, allowed for the design of a controlled drug release system using the HNT-medicine intercalation compounds (i.e., drug delivery systems) [23,24,25,26].

The aim of this paper was to determine:

- The effect of HNT additions on the mechanical and biological properties of the as-produced thermoplastic linear polyurethane-based nanocomposites;
- Compatibility of nanocomposite synthesis with 3D printing methods for personalized intervertebral disc prostheses applications.

**Figure 1:**
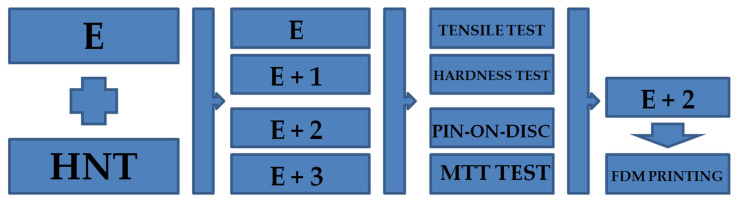
Scheme of the research methodology.

Nanocomposites were produced using a twin-screw extruder. HNT was introduced into the TPU matrix to produce nanocomposites with higher expected mechanical properties (Young’s modulus, fracture stress, and elongation stress) compared with pure TPU. HNT additions were also expected to increase the abrasion resistance. The produced nanocomposites must also be completely non-toxic to normal human cells. Fabricated nanocomposites with a confirmed structure were then microscopically made into flasks using an injection method and filament for 3D printing. Injected samples were then tested using the static tensile test, hardness test, pin-on-disc, and cytotoxicity test MTT while printed samples were subjected to tensile strength tests. The 3D printing of these materials provides many advantages, including unique tailoring to a patient’s needs, which is in line with the current trend of personalized medicine [27,28]. The conducted preliminary tests, including plate-tubular halloysite, proved that twin-screw extrusion provides nanocomposites with improved mechanical properties compared with the original matrix with no toxicity to normal cells [29]. The scheme of the research methodology is shown in Figure 1.

## 2. Materials and Methods

### 2.1. Materials

**Figure A1:**
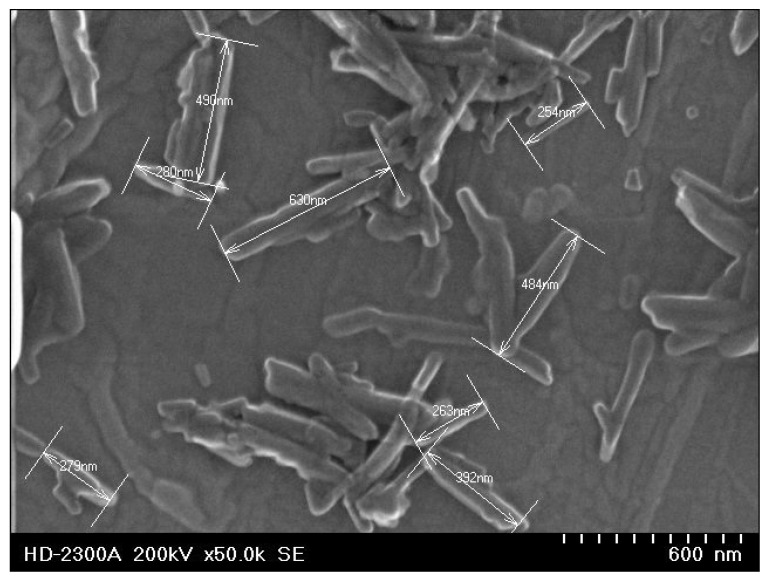
STEM images of HNT.

Synthesized nanocomposites were based on thermoplastic linear polyurethane filled with halloysite nanotubes (HNT). Elastollan 1185A, a polyether-based, linear thermoplastic polyurethane linear (TPU) was made by BASF. Elastollan 1185A was chosen as the model material to illustrate the effects of HNT additions on the composite properties. In addition, Elastollan 1185A is a polyether-based polyurethane that is insoluble in water, the environment of the human body. As a result, implants made of this type of polyurethane will not undergo rapid hydrolytic degradation. HNT was purchased from Sigma Aldrich (Saint Louis, MO, USA). Halloysite nanoclay has a tube morphology with a 30–70 nm diameter and 1–3 µm length. STEM imaging of HNT is presented in Figure A1 in Appendix A.

Prior to extrusion, HNT was subjected to a hydrophilicity reduction. Modifications were carried out according to those escribed by Mrówka et al. [29]. Firstly, the cation exchange capacity (CEC) of the mineral material was determined using the cobalt (III) hexamine method. The measured CEC was equal to 8.02 meq/100 g. Secondly, the material was modified using hexadecyltrimethylammonium bromide (HDTMA–Br) in an amount equal to 1 CEC. For this, an aqueous suspension of HNT was prepared, and HDTMA-Br was introduced in the form of an aqueous solution after sonification. The formed suspension was stirred for 24 h, followed by centrifugation and drying at 70 °C for 24 h. 

### 2.2. Preparing of the Nanocomposites 

Prior to the extrusion process, Elastollan 1185A was dried in a convection oven (POL-EKO-APARATURA, Wodzisław Śląski, Poland) for three hours at 110 °C. The nanocomposites were prepared using a Leistritz ZSE 27 HP twin-screw extruder (Leistritz AG, Nuremberg, Germany). Synthesized nanocomposites were based on thermoplastic polyurethane filled with modified HNT weight fractions of 1, 2, and 3% to produce the following materials: [E + 1], [E + 2], and [E + 3]. The nanocomposite extrusion parameters consisted of 100–175 °C zone temperatures, 180 °C mass temperature, 45 bar pressure, 270 RPM screw rotation, and 12 kg/h efficiency. All test samples were prepared by injection molding used an Arburg Allrounder 270–210–500 machine (ARBURG GmbH, Loßburg, Germany). The temperature was 180–190 °C with 90 MPa pressure. All tests were carried out at room temperature (22 °C) and 50% humidity.

### 2.3. Extrusion Molding [E + 2] Material into a Filament Used in 3D Printing

The experimental results showed that the resulting nanocomposites exhibit improved mechanical properties (higher tensile modulus, higher fracture stress, higher fracture elongation) compared with the polyurethane matrix material. The formed composites were characterized by lower wear compared with the parent TPU matrix while still possessing similar hardness. All tested compounds show no cytotoxicity towards normal human cells.

Among the tested materials, nanocomposites containing 2 wt.% HNT [E + 2] were selected for the next research stage. This is due to the fact that these samples showed the highest fracture elongation and lowest abrasion among the tested materials. These nanocomposites measured similar hardness with no cytotoxicity to normal human cells. Ultimately, [E + 2] will be used in the production of personalized soft tissue prostheses. Therefore, it is necessary to evaluate its use in further stages of additive technologies. Among the available additive technologies, the most popular technology is fused deposition modeling (FDM), which involves printing with a liquid thermoplastic. This technology, thanks to its simplicity, offers high speed printing of the desired elements using cheap and easily available printers to obtain personalized prostheses and implants in a short timeframe.

Prior to extrusion, the granular form of the nanocomposite [E + 2] was dried in a laboratory dryer for three hours at 110 °C. The filament extrusion process was carried out using a single-screw extruder built independently by Noctuo employees. Extrusion parameters for [E + 2] were as follows: zone temperature: 151–191 °C, mass temperature: 210 °C, pressure: 36 bar, screw rotation: 15 RPM, capacity: 20 kg/h. As a result of the extrusion process, a filament with a diameter of 1.75 ± 0.1 mm was obtained. From the FRM-printed material, samples with dimensions equal to those obtained with the injection technology were printed. The print properties were as follows: samples adjacent a larger surface to the working table, layer height: 0.15 mm, degree of filling: 100%, filling type: outline. The optimal temperature for printing was 230 °C, and the optimum printer temperature was 55 °C.

### 2.4. Modification of the Printer to Enable Printing on it from Thermoplastic Elastomers

A 3DGence One printer (3DGence, Przyszowice, Poland) was used for experiments. Printers of this type are not adapted to printing from thermoplastic elastomers. Ultimately, the manufacturer intended them to be printed with PLA and ABS. However, in order to print the [E + 2] material, the printer was modified, making it possible to obtain test samples made of the tested elastic material. Changes made in the printer are presented in Table 1.

### 2.5. Scanning Transmission Electron Microscopy (STEM)

The structure and morphology of the nanocomposites were characterized using scanning transmission electron microscopy (STEM). For STEM observations, specimens were prepared using a focused ion beam (FIB) technique using a SEM/Ga-FIB Helios NanoLab™ 600i microscope (FEI, Hillsboro, OR, USA). STEM measurements were completed using a S/TEM TITAN 80–300 microscope equipped with an energy-dispersive X-ray spectrometer (EDS). High angle angular dark field (HAADF) images were collected using a 24.5-mrad probe semi-angle. The HAADF detector range was 47–200 mrad.

### 2.6. Tensile Test 

**Figure 2:**
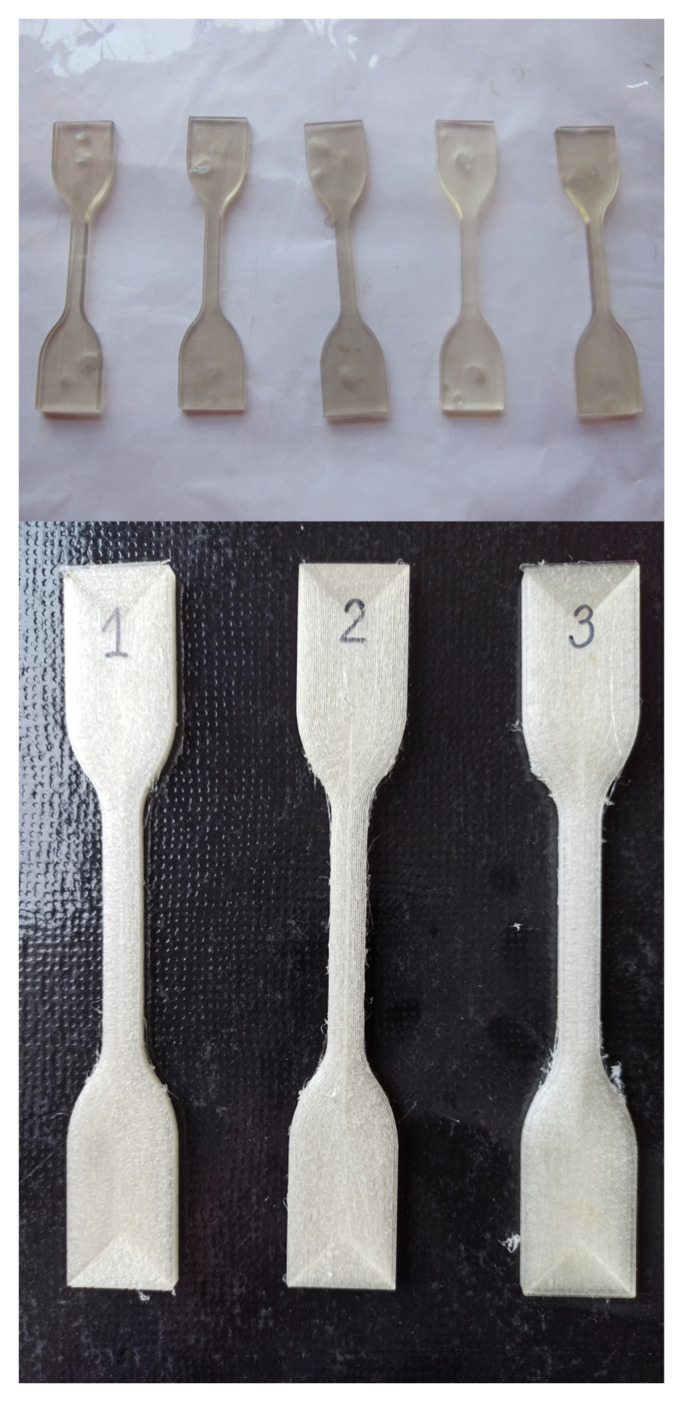
Injected and printed samples.

**Figure A2:**
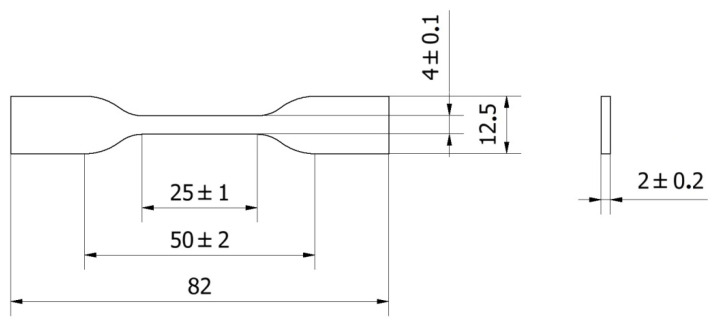
Sample geometry.

The tensile strength test was measured in accordance with EN ISO 527–1 on a tensile machine Instron 4465 (Instron, Norwood, MA, USA) equipped with a mechanical contact extensometer [30]. The test speed was 50 mm/min. The sample geometry is shown in Figure A2. A sample population of 5 was used for all experiments. The injected and printed samples are shown in Figure 2. Using the tensile test results for each of the tested sample populations, the fracture stress and elongation were determined along with the tensile modulus.

### 2.7. Shore a Hardness Test 

Hardness measurements of the tested materials were carried out using a hardness durometer Shore A type Zorn (Zorn Instruments GmbH & Co., Hansestadt, Germany). The Shore A hardness test was completed in accordance with ISO 686 [31]. Five measurements were taken for each composite while maintaining a distance of at least 10 mm from the sample edge and between individual measurements.

### 2.8. Pin–on–Disc Abrasion Test

Abrasion testing was performed using a Tribometer CSM Instrument (Needham, MA, USA) in accordance with ASTM G99 [32]. Test samples were prepared in the form of 10 mm × 10 mm × 4 mm cuboids. Prior to testing, samples were cleaned with technical ethanol. The ball moving after the sample with a 6 mm dimension was made of zirconium dioxide. The ball was pressed against the sample with a force of 5 N and linear speed of 10 cm/s. The abrasion for the tested materials was defined as the change in the coefficient of friction (µ) on the 100 m road.

### 2.9. MTT Cytotoxicity Test

Cytotoxicity testing of materials was carried out using human epidermal keratinocytes (HaCaT; Cat# 300493, RRID: CVCL_0038) purchased from CSL Cell Line Service GmbH (Eppelheim, Germany). Cell viability was assessed via MTT test (3–[4,5–dimethylthiazol–2–yl]–2,5–diphenyltetrazolium bromide). Before placing in the cell culture, material samples were washed with methanol (96%) and then sterilized with UV light for 24 h. Cells were inoculated in Petri dishes at a concentration of 10^5^ cells per well. Cell cultures were implemented with test materials and incubated for 24 and 72 h, respectively, at 37 °C in a humidified atmosphere saturated with 5% CO_2_. After the specified time, the culture medium was removed and replaced with trypsin cell collection solution. Following trypsin neutralization, the cell suspension was centrifuged (2000 RPM, 3 min, room temperature), and the cell pellet was resuspended in MTT solution (50 µL, 0.5 mg/mL in RPMI 1640 without phenol red, Sigma-Aldrich (St. Louis, MO, USA)). After 3 h of incubation, the MTT solution was removed, and the resulting formazan was dissolved in isopropanol: HCl. Absorbance at 570 nm was measured spectrophotometrically using a plate reader. The experiment was carried out in three independent replicates.

## 3. Results and Discussion 

### 3.1. Scanning Transmission Electron Microscopy (STEM)

**Figure 3:**
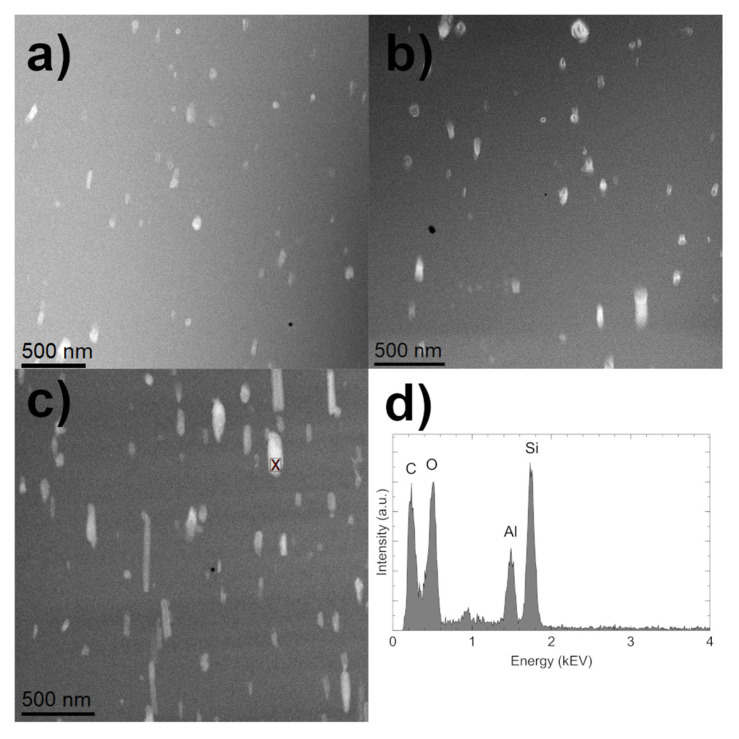
STEM-HAADF images of nanocomposites: E + 1 (**a**), E + 2 (**b**), E + 3(**c**). Energy dispersive spectra (EDS) obtained for single halloysite tube in polyurethane matrix at the point indicated in Figure (**d**).

A comparison of the morphology of the three tested composites is visible in Figure 3.

These results show that the HNTs (bright tubular region) are evenly embedded within the polymer matrix (dark region). The components of the composite are easily distinguished due to the use of the high-angle annular dark field (HAADF) detector, which registers electrons passing through the sample and scattered at a high angle (Rutherford scattering). As a result, the recorded signal intensity is proportional to the atomic number Z of the dominant element in a given specimen region, providing Z-contrast imaging. As halloysite nanotubes contain additional Al and Si (Figure 3d), both of which have higher Z values compared with the matrix material elements (mainly C and O), they are imaged more brightly than the surrounding polymer matrix [33]. The STEM images confirm that most of the HNTs are homogeneously dispersed. The STEM image is a two-dimensional projection of the three-dimensional composite structure. Although the sample was prepared in the form of a thin foil (~100 nm thickness), the preferred nanotube orientation is noticeable for all the investigated materials. In the presented images, the nanotubes display a vertical orientation. The HNTs’ length does not exceed 500 nm, and the diameter does not exceed 100 nm. Figure 3a–c also confirms the increasing concentration of halloysite nanotubes in the manufactured composites.

### 3.2. Tensile Test 

**Figure 4:**
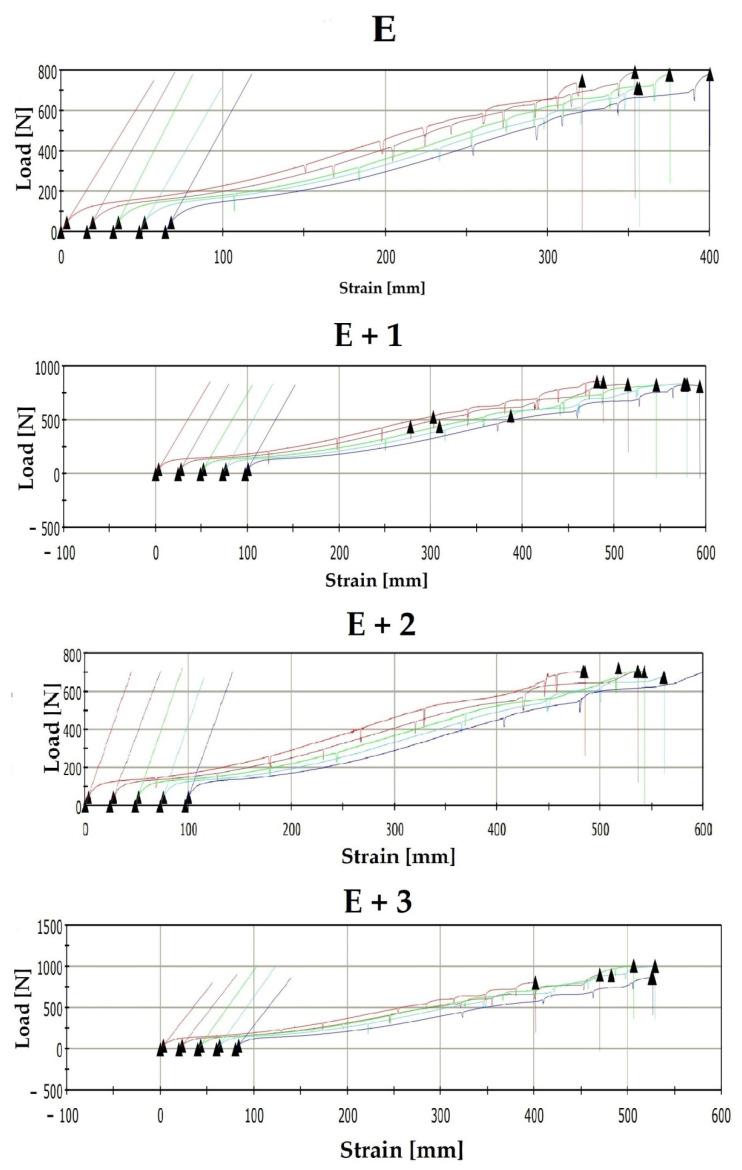
The load–strain curves of the materials.

Static tensile tests were performed on samples made of unmodified TPU [E] and the synthesized nanocomposites [E + 1], [E + 2] and [E + 3]. The load-strain curves of the materials are shown in Figure 4.

**Figure 5:**
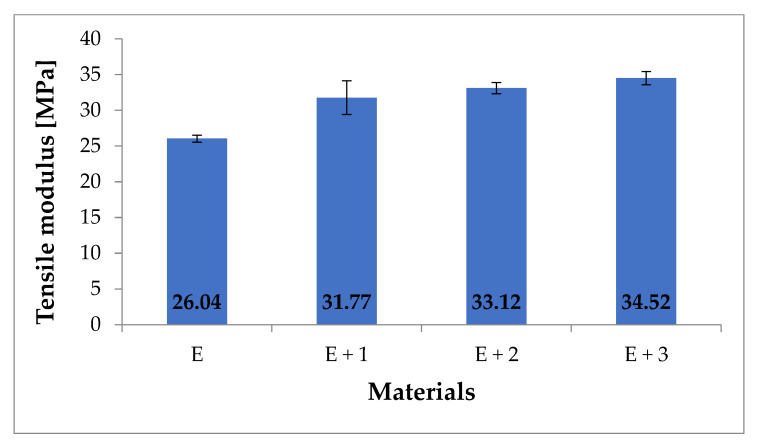
Tensile modulus for tested materials (MPa).

**Figure 6:**
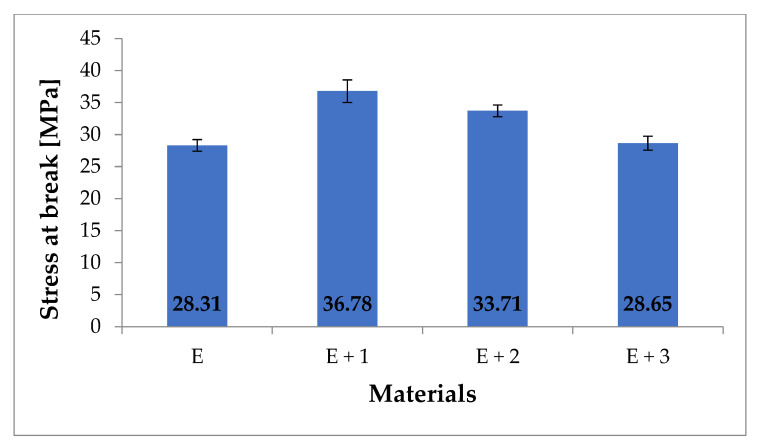
Stress at break for tested materials (MPa).

**Figure 7:**
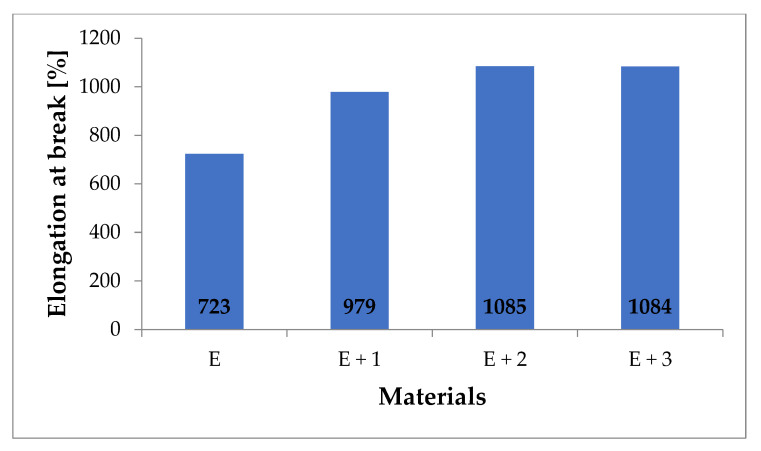
Elongation at break for tested materials (%).

The materials were characterized by measuring the tensile modulus (Figure 5), fracture stress (Figure 6), and fracture elongation (Figure 7).

The mechanical testing results for these materials indicate that the tensile modulus increases with increasing HNT concentration. For unmodified TPU [E], the measured tensile modulus is 26.04 MPa while for [E + 1] an increase of 22% (31.77 MPa) is measured. For the [E + 2] nanocomposite, the tensile modulus increase compared to [E] is 27% (33.12 MPa) while for [E + 3] an increase of 33% (34.52 MPa) was noted. The obtained results do not differ significantly from those presented in the literature. The publication [34] reported a 32% increase in tensile modulus for nanocomposites containing 1% HNT mass content. Similarly, the publication [35] reported a 40% increase in tensile modulus for nanocomposites containing 3.7% HNT. The obtained tensile modulus values show that as the amount of filler in the nanocomposite increases, so does the rigidity.

For the nanocomposite [E + 1], a 30% increase in the fracture stress compared with [E] was measured (i.e., 28.31 MPa compared with to 36.78 MPa). For [E + 2], a 20% increase compared with [E] (i.e., 28.31 MPa vs. 33.71 MPa) was also read, but this measured value was 10% lower than the fracture stress for [E + 1]. The fracture stress for [E + 3] (28.64 MPa) is comparable with the value measured for unmodified TPU. The literature reports similar relationships for nanocomposites filled with some form of HNT. For instance, the publication [36] showed an increase in fracture stress by 37% for a nanocomposite containing 1% HNT. The work in [34] describes a 44% increase in stress at break also for a nanocomposite with 1% HNT content. Similarly, the authors of [37] presented studies in which a nanocomposite containing 1% HNT showed a 43% increase in fracture stress compared with the TPU matrix. Additionally, 2% additions of HNT to the TPU matrix have been reported to increase the fracture stress by 26% [38].

[E + 1] showed a 35% increase in elongation prior to fracture compared with [E] (i.e., 723 to 979%) while [E + 2] showed a 50% increase in elongation compared with the matrix (i.e., 723 to 1085%). For [E + 3], the elongation prior to fracture was 1084%, which is identical with [E + 2]. The elongation at break values available in the literature have significantly higher values than those described in this paper. In [37], a 1% HNT-TPU nanocomposite resulted in 144% increase in elongation prior to fracture compared with native TPU. In publications studying similar nanocomposites with 2% HNT content, 67% [33] and 100% [38] increased fracture elongation was measured compared to TPU. The differences in these results can be attributed to the adopted method of producing nanocomposites or by the materials used in the study. The methods described in the publications are laboratory methods in which processes for obtaining nanocomposites are carried out on a small scale while this paper relies on industrial production methods (i.e., twin–screw, 10–zone extrusion). The source materials also play a large role. The literature describes nanocomposite production using commercially available TPU while the source materials for this study use chemically modified TPU to reduce hydrophobicity and improve HNT adhesion [34,35,36,37,38].

### 3.3. Shore A Hardness Test

**Figure 8:**
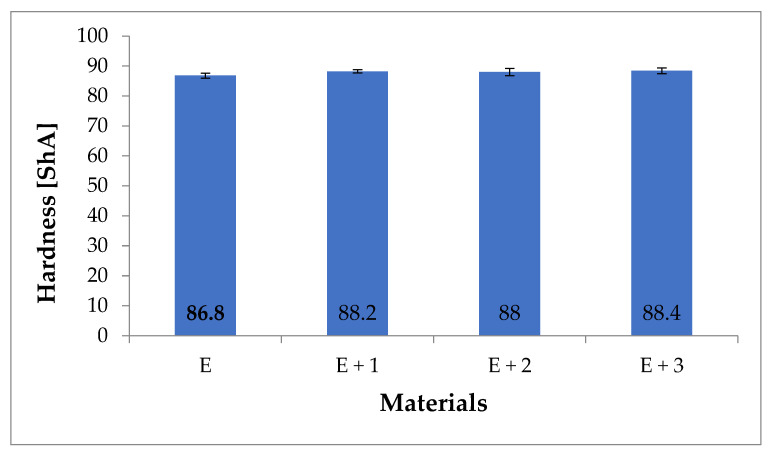
Hardness values for tested materials (ShA).

The hardness values for the composites are presented in Figure 8.

The hardness of unmodified TPU was measured at 86.8 ShA, and the measured hardness of the synthesized nanocomposites were as follows: [E + 1] 88.2 ShA, [E + 2] 88 ShA and [E + 3] 88.4 ShA. For the nanocomposites, a slight ~2% increase in hardness was observed compared to native TPU. However, considering the accuracy of the Shore A hardness test, it can be concluded that HNT additions to the TPU matrix does not significantly impact hardness.

### 3.4. Pin–on–Disc Abrasion Test

**Figure 9:**
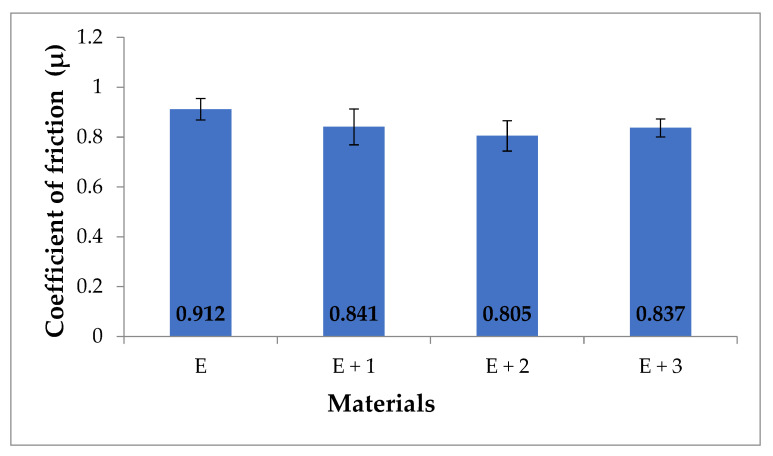
Coefficient of friction (µ) for tested materials.

The average results of friction coefficients (µ) for the tested materials are shown in Figure 9.

Changes in the friction coefficient reflect the changes in motion resistance, because the friction coefficient is proportional to the value of the friction force. The measured friction coefficients of the samples are as follows: [E] was 0.912, [E + 1] was 0.841, [E + 2] was 0.805, and [E + 3] was 0.837. For all tested nanocomposites, a decrease in wear was observed in relation to [E]. The largest decrease in the friction coefficient was observed for [E + 2], which was 13% in relation to [E]. The friction coefficient values for the samples [E + 1] and [E + 3] were essentially the same. When particle size is reduced to the nano-scale, the wear performance of composites may be significantly different from that of micro-particle filled systems. Agglomeration is a common problem in polymer nanocomposites, especially at higher nanofiller contents. In most cases, optimum inorganic particle filler contents could be identified at which the highest wear resistance of these polymers occurred. The optimum filler content of inorganic particles was mostly in a range between 1 and 6 vol% [39,40]. The inorganic compound halloysite (Al_4_[Si_4_O_10_](OH)_8_·4H_2_O) introduced into TPU in 1–3 mass % reduces the coefficient of friction with respect to the TPU matrix.

### 3.5. MTT Cytotoxicity Test

**Figure 10:**
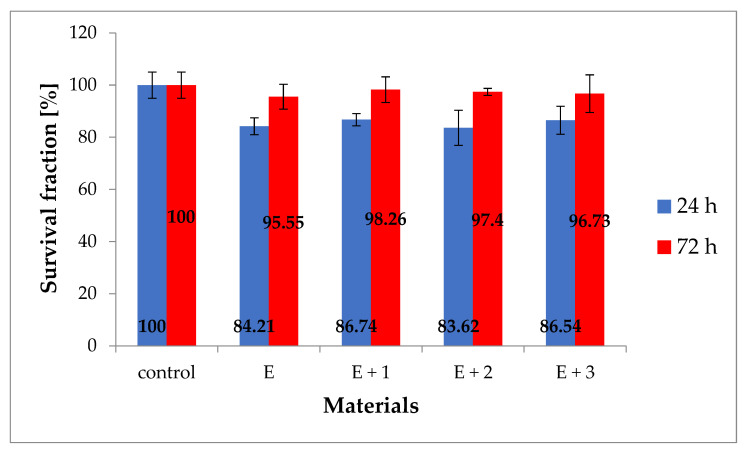
Cytotoxicity of the tested materials relative to the HaCaT line (%).

The cytotoxicity results of the tested materials against the HaCaT line after 24 and 72 h are shown in Figure 10. The purpose of the test was to assess the toxicity of the synthesized materials for potential use as biomaterials. Toxicity has been defined as a material property that causes a disorder or death of human cells. These changes are primarily seen in abnormal cell metabolism, and the degree of this phenomenon can be assessed using the MTT test. The results are presented in the form of graphs of the survival fraction (%) on the incubation time of cells with the tested material.

After 24 h for the synthesized materials [E], [E + 1], [E + 2] and [E + 3], the average cell viability was 84%, 87%, 83%, and 86%, respectively. After 72 h of incubation, the same materials had a viability of 95%, 98%, 97%, and 96% of the population relative to the control (100%). The presented results show a decrease in the fraction of live cells in relation to the control sample during the first day, and then reconstitution of the level of live cells similar to the level of the control sample after 72 h of incubation. The decrease in viability during the first 24 h can be explained by cellular stress caused by the appearance of a foreign element in the medium (i.e., the sample being tested) and the partial mechanical damage to a number of cells during sample implementation in culture. After 72 h, the number of cells in culture increases, which can be explained by the disappearance of cellular stress and subsequent cellular growth, which in laboratory conditions provided favorable conditions for proliferation. The obtained test results clearly indicate the lack of cytotoxicity of the tested materials towards normal human body cells of the HaCaT line at the standard time of the MTT test.

### 3.6. Comparison of Mechanical Properties of Injected Samples with Printed Samples

**Figure 11:**
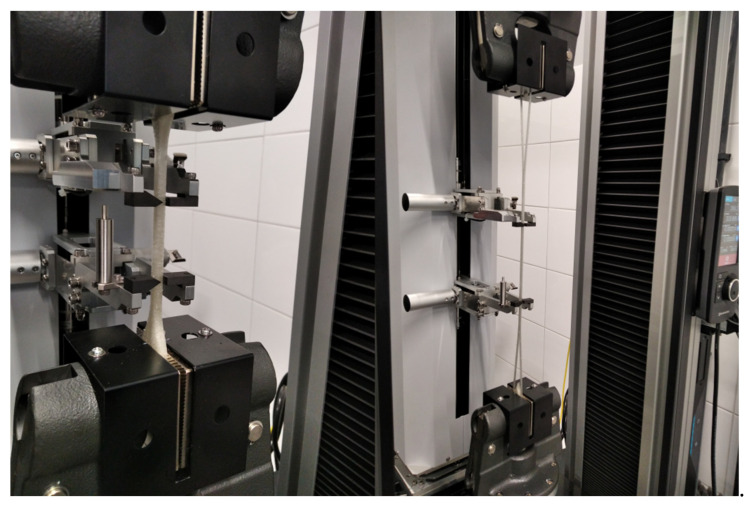
Printed sample before and during the static tensile test.

After printing, the samples were conditioned according to the standard presented in [37] and then stretched with parameters consistent with injected samples. The sample morphology before and during stretching is shown in Figure 11. The results comparing the mechanical properties of the printed and injected samples are summarized in Table 2.

The results presented in Table 2 show that the printed samples are characterized by inferior mechanical properties compared with injected samples. All measured parameters were lower for samples printed using FDM printing. In the case of the tensile modulus, the value decreased by 1/3 compared to the samples produced via injection. The measured fracture stress was 71% lower for printed samples compared with injection samples. Regarding elongation prior to fracture, there was a decrease of 65% for the samples synthesized using FDM printing. The difference in mechanical properties can be attributed to the sample preparation methods. Samples produced by the injection method are uniform throughout their structure whereas samples prepared via 3D printing consist of strands of material which are distributed with each other by means of the printer’s extruder. As a result, the resultant materials do not have a uniform structure due to the presence of spaces between successive material layers. In order to eliminate the differences between printed and injected samples, it is necessary to change the printing parameters to obtain samples with improved mechanical properties. Another idea is to use a professional printer for sample printing from thermoplastic elastomers for the production of samples.

## 4. Conclusions

Tested nanocomposites with variable HNT filler concentration with in a TPU matrix show improved mechanical and tribological properties compared with native TPU. Nanocomposites with a filler content of 1 to 3 wt.% measured higher tensile modulus, fracture stress, and elongation prior to failure compared with pure TPU. The new nanocomposites measured similar hardness to native TPU. All nanocomposites showed a lower friction coefficient than TPU. It was confirmed that the synthesized nanocomposites do not have a toxic effect on normal cells. Out of the three nanocomposite compositions, [E + 2] (2 wt.% HNT in TPU) showed the best physical properties. An attempt to conduct [E + 2] into a filament that can be used in 3D printing using FDM technology to produce personalized implants and prostheses was made. Samples obtained via 3D printing, however, were characterized by inferior mechanical properties compared with nanocomposite samples manufactured by injection. This is primarily due to the manufacturing method itself. Discontinuities formed between the individual layers of the material produce notch effects and thus diversify the mechanical properties directionally.

## Figures and Tables

**Table 1:** Changes made to the 3DGence One printer.

Modification	Purpose of Modification
Enlargement of the printer with a closed chamber	Maintaining a constant temperature inside the printing chamber allows for higher quality printouts, especially for long-lasting prints. The variable temperature of the environment depending on the time of day may reduce the adhesion of subsequent layers of the filament to each other, which may result in worse physical properties of the printout. The chamber is made of Plexiglass—a transparent material that is characterized by a low heat conduction coefficient.
Gravitational drop of filament	In the printer undergoing modernization—the prototype of the 3DGence One printer, the filament holder is located on the side of the housing, below the extruder. When 3D printing from TPU material, an important modification is the appropriate location of the spool. The assumption is that the filament that is pulled through the extruder knob into the nozzle is an assumption, however due to the flexibility of the TPU it may stretch causing incomplete extrusion. The solution to this problem is to position the spool with the filament above the printer, allowing the material to be fed vertically. Thanks to this solution, the resistance to movement is lower.
Adding an element around the knurl to prevent buckling of the filament	During test prints of flexible materials on the 3DGence One prototype, there was a problem with forgiving the filament near the knurling. The optimal solution consists in making a plate of aluminum sheet with dimensions of approximately 10 mm × 6 mm × 3 mm, which will be glued to a fixed element located under the knurl.

**Table 2:** Comparison of mechanical properties of injected and printed samples of [E + 2] material.

Parameters	Injected Samples	Printed Samples
Tensile modulus [MPa]	33.12	22.01
Stress at break [MPa]	33.71	9.75
Elongation at break [%]	1085	383

## Data Availability

Not applicable.
